# Brain Ceramide Metabolism in the Control of Energy Balance

**DOI:** 10.3389/fphys.2017.00787

**Published:** 2017-10-12

**Authors:** Céline Cruciani-Guglielmacci, Miguel López, Mélanie Campana, Hervé le Stunff

**Affiliations:** ^1^Unité de Biologie Fonctionnelle et Adaptative, Centre National de la Recherche Scientifique, Unité Mixte de Recherche, Université Paris Diderot, Université Sorbonne Paris Cité, Paris, France; ^2^Department of Physiology, Center for Research in Molecular Medicine and Chronic Diseases (CiMUS), Instituto de Investigación Sanitaria de Santiago de Compostela, Universidade de Santiago de Compostela, Santiago de Compostela, Spain; ^3^UMR9197 Institut des Neurosciences Paris Saclay (Neuro-PSI), Université Paris-Saclay, Saclay, France

**Keywords:** hypothalamus, lipid sensing, lipotoxicity, ceramides, energy homeostasis

## Abstract

The regulation of energy balance by the central nervous system (CNS) is a key actor of energy homeostasis in mammals, and deregulations of the fine mechanisms of nutrient sensing in the brain could lead to several metabolic diseases such as obesity and type 2 diabetes (T2D). Indeed, while neuronal activity primarily relies on glucose (lactate, pyruvate), the brain expresses at high level enzymes responsible for the transport, utilization and storage of lipids. It has been demonstrated that discrete neuronal networks in the hypothalamus have the ability to detect variation of circulating long chain fatty acids (FA) to regulate food intake and peripheral glucose metabolism. During a chronic lipid excess situation, this physiological lipid sensing is impaired contributing to type 2 diabetes in predisposed subjects. Recently, different studies suggested that ceramides levels could be involved in the regulation of energy balance in both hypothalamic and extra-hypothalamic areas. Moreover, under lipotoxic conditions, these ceramides could play a role in the dysregulation of glucose homeostasis. In this review we aimed at describing the potential role of ceramides metabolism in the brain in the physiological and pathophysiological control of energy balance.

## Hypothalamic lipid metabolism: a basic pathway regulating energy balance

The hypothalamus regulates a vast number of homeostatic functions. Among them, regulation of endocrine axes, reproductive function, and energy balance are of particular importance (Williams et al., 2001; King, 2006). Despite the well-established role of neuropeptides, several lines of evidence have demonstrated that modulation of hypothalamic lipid metabolism is a very important mechanism regulating energy balance. Indeed, while neuronal activity primarily relies on glucose, the brain expresses at high level enzymes responsible for the transport, utilization and storage of lipids. Since the work of Oomura et al. (1975), growing body of evidence suggests that fatty acids (FA) are able to modulate neuron activity in hypothalamus and regulate energy balance through the control of insulin secretion, hepatic glucose production, adipose storage and food intake (Obici et al., 2002; Cruciani-Guglielmacci et al., 2004; Lam et al., 2005). This phenomenon has been called “lipid sensing,” and the molecular mechanisms involved are still matter of controversy. It includes plasma membrane proteins such as G-protein coupled receptor 120 (GPR120) or FA translocase (FAT/CD36), but also intracellular events including FA oxidation or synthesis of diacyl-glycerol (DAG) and ceramides (Magnan et al., 2015). In addition lipid membrane composition itself may regulate neuronal signaling pathways as the lipid profile in specific microdomains named lipid rafts (enriched in cholesterol, saturated phospholipids and sphingolipids) could modulate the signaling pathway integration through changes in the affinity of proteins to concentrate in these domains (Yaqoob and Shaikh, 2010). Interestingly, key enzymes involved in FA synthesis and oxidation, namely acetyl-CoA carboxylase (ACC), fatty acid synthase (FAS), malonyl-CoA decarboxylase (MCD) and carnitine palmitoyltransferase 1 (CPT1) are expressed at high levels in the arcuate (ARC), paraventricular (PVH), dorsomedial (DMH), and ventromedial (VMH) nuclei, which are, with the lateral hypothalamic area, among the most relevant hypothalamic sites modulating energy homeostasis (Dowell et al., 2005; Lopez et al., 2007; Gautron et al., 2015). AMP-activated protein kinase (AMPK), a cellular energy sensor that modulates FA metabolism by controlling ACC and MCD activities and FAS expression, is also highly expressed in the hypothalamus (Lage et al., 2008; Carling et al., 2011; Hardie et al., 2012; Lopez et al., 2016).

In addition to this anatomical data, physiological, pharmacological and genetic evidence has shown that the modulation of these activities at hypothalamic level impacts energy homeostasis. Thus, treatments with FAS inhibitors, such as cerulenin and C75 (Loftus et al., 2000; Hu et al., 2003), and with factors that decrease FAS expression, such as leptin, tamoxifen, and estradiol (Lopez et al., 2006; Wolfgang et al., 2007; Martinez de Morentin et al., 2015), as well as the specific ablation of hypothalamic FAS (Chakravarthy et al., 2007) induce a remarkable weight loss and hypophagic effect, which depends on accumulation of malonyl-CoA (the product of ACC and the substrate of FAS) in the hypothalamus. Of note, this anorectic action is linked to decreased expression of orexigenic (AgRP and NPY) neuropeptides and elevated expression of anorexigenic (CART, POMC) ones in the ARC (Loftus et al., 2000; Hu et al., 2003; Lopez et al., 2006; Chakravarthy et al., 2007; Wolfgang et al., 2007). One interesting possibility to explain this action is the inhibitory effect of malonyl-CoA on CPT-1a, therefore preventing the access of long-chain fatty acyl-CoAs to the mitochondria and leading to its cytoplasmic accumulation which would be sensed as a signal of nutrient abundance. This idea is supported by the fact that genetic ablation of hypothalamic CPT-1a activity reduces food intake (Obici et al., 2003; Wolfgang et al., 2006, 2008).

Hypothalamic AMPK plays a major role in the modulation of both feeding (Andersson et al., 2004; Minokoshi et al., 2004; Claret et al., 2007; Andrews et al., 2008; Lopez et al., 2008, 2016) and energy expenditure, specifically through the control of hormone-induced brown adipose tissue (BAT) thermogenesis. Specifically, within the VMH, a decreased AMPK activity activates BAT thermogenesis through increased sympathetic nervous system (SNS) outflow. Notably, this pathway, initially described for central effects of thyroid hormones on energy balance (Lopez et al., 2010), is also shared by leptin (Tanida et al., 2013), BMP8B (bone morphogenetic protein 8B) (Whittle et al., 2012; Martins et al., 2016), estrogens (Martinez de Morentin et al., 2014, 2015), glucagon-like-peptide 1 agonist (Beiroa et al., 2014) and nicotine (Martinez de Morentin et al., 2012; Seoane-Collazo et al., 2014). Finally, we proposed the VMH AMPK-SNS-BAT axis as a canonical mechanism modulating energy homeostasis (Lopez et al., 2013, 2016; Contreras et al., 2015).

## Hypothalamic lipotoxicity: a pathophysiological mechanism of obesity

In peripheral tissues, accumulation of reactive lipid species, such as DAG, free fatty acids, free cholesterol, and ceramides is a pathogenic mechanism of insulin resistance, type 2 diabetes, liver and cardiovascular disease (Chaurasia and Summers, 2015). This lipotoxicity occurs through inflammation and endoplasmic reticulum (ER) stress (Ozcan et al., 2004; Martinez de Morentin and Lopez, 2010; Unger et al., 2010; Virtue and Vidal-Puig, 2010; Bellini et al., 2015), which, of note, can also occur in the central nervous system (CNS), as observed in certain neurodegenerative disorders (i.e., polyglutamine diseases, Parkinson's disease and amyotrophic lateral sclerosis) (Ilieva et al., 2007). In particular previous studies have demonstrated that ER stress and activation of the unfolded protein response played a key role in promoting insulin resistance in peripheral tissues (Kammoun et al., 2009). In the hypothalamus, ER stress also induces insulin resistance, and leptin resistance, leading to weight gain (Zhang et al., 2008; Ozcan et al., 2009). Moreover, a chronic lipid excess condition, such as overweight and obesity, has been shown to impair lipid sensing, and this deregulation—namely brain lipotoxicity—may contribute to the setting of type 2 diabetes in predisposed subjects through changes in autonomic nervous system activity (Picard et al., 2014a). However, one key question that remains to be addressed relates to the status of lipid metabolism and whether accumulation of specific lipid species occurs in the hypothalamus. Recent studies point out that ceramides accumulation under lipotoxic conditions could play a role on the deregulation of energy balance in both hypothalamic and extra-hypothalamic areas (Le Stunff et al., 2013; Contreras et al., 2014; Picard et al., 2014b).

## *De novo* ceramide biosynthesis in brain

In peripheral organs, ceramides are important mediators of lipotoxicity: they accumulate in insulin-sensitive tissues and in pancreatic β cells during the development of obesity, and their intracellular levels correlate with both insulin resistance and β cell apoptosis (Bellini et al., 2015). In rodents, it has been demonstrated that enzymes of *de novo* ceramides synthesis are expressed in hypothalamus and hippocampus (Contreras et al., 2014; Picard et al., 2014b).

**Figure 1:**
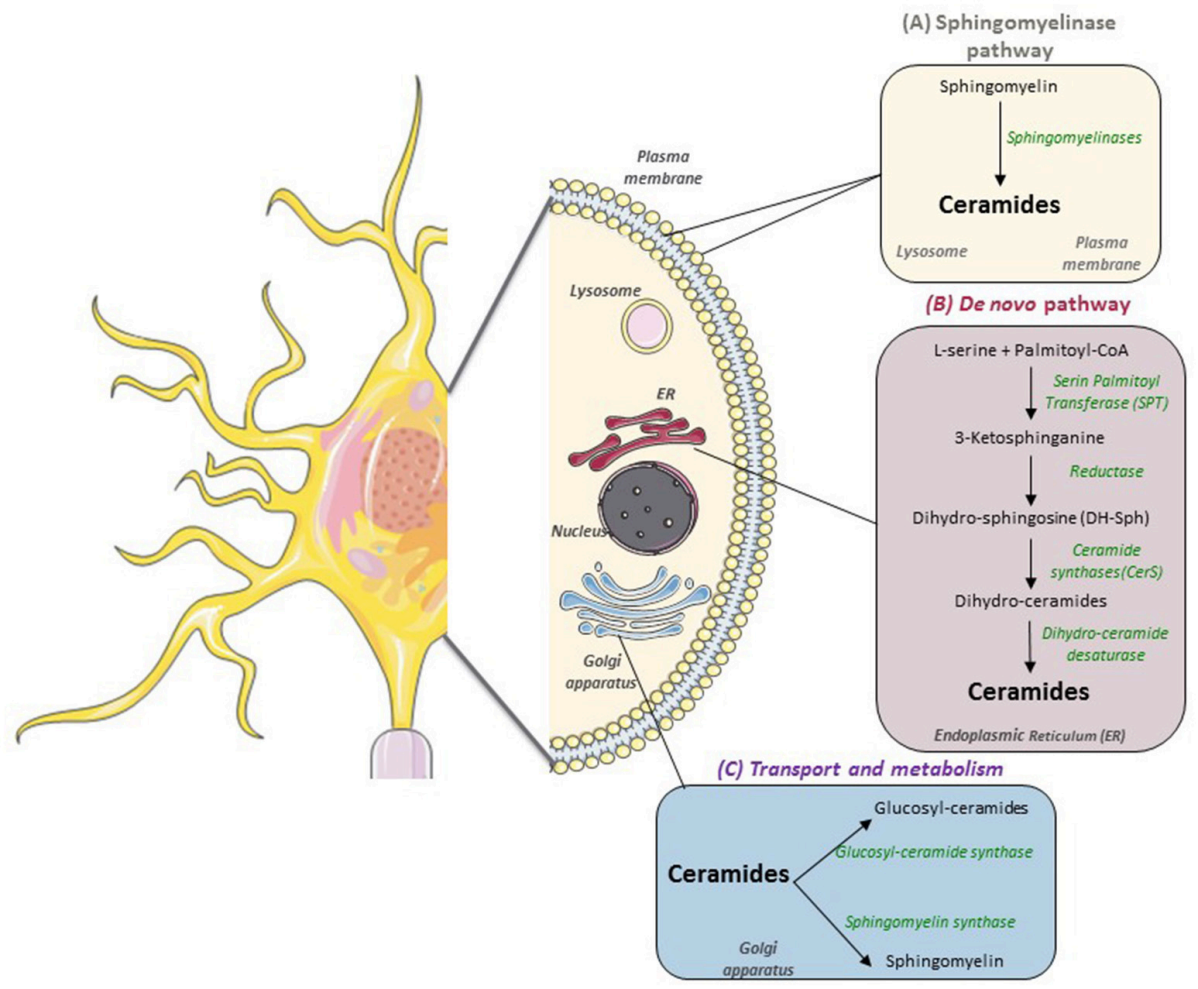
Sphingolipids metabolism in nervous cells. In mammals, there are two main pathways to produce sphingolipids: **(A)** the catabolic sphingomylinase pathway that takes place in the lysosomal and plasma membranes and leads to the degradation of sphingomyelin (SM) into ceramides by Sphingomyelinases (SM); **(B)** the *de novo* synthesis pathway which starts on the cytoplasmic face of the endoplasmic reticulum (ER) with the condensation of Palmitoyl-CoA and L-Serine to form 3-ketosphinganine. **(C)** Then, ceramides are transported to the Golgi apparatus to be metabolized into more complex sphingolipids such as glucosyl-ceramides and sphingomyelin.

In the context of obesity-associated lipid excess, *de novo* ceramides are mainly produced from saturated FA such as palmitate, and this synthesis begins in the cytoplasmic face of the ER (Figure 1). The first step is the condensation of L-serine with palmitoyl-CoA to form 3-ketosphinganine, catalyzed by serine palmitoyl-transferase (SPT) (Hannun and Obeid, 2008). Then 3-ketosphinganine is reduced to dihydrosphingosine (DH-Sph) by 3-ketosphinganine reductase and the resulting DH-Sph acts as a substrate for ceramide synthases (CerS), leading to the production of dihydroceramides. In mammals, six CerS isoforms are expressed, they have distinct specificities depending on the acyl-CoA chain length they use for N-acylation of DH-Sph (Pewzner-Jung et al., 2006; Mullen et al., 2012) Dihydro-ceramides are transformed into ceramides by the dihydroceramide desaturase DES1 (Causeret et al., 2000). Ceramides are then transported to the Golgi apparatus where they are converted into sphingomyelin or into glucosyl-ceramides by sphingomyelin synthase and glucosyl-ceramide synthase, respectively (Hanada et al., 2003).

Glucosylceramide synthase (GCS) derived gangliosides are acidic glycosphingolipids that are prominently expressed by neurons (Jennemann et al., 2005). They contribute to the formation of membrane microdomains which regulate intracellular signal transduction (Simons and Gerl, 2010). In particular, Nordström et al. have recently demonstrated that adequate function of the hypothalamic leptin receptor (ObR) requires GCS expression (Nordstrom et al., 2013).

In addition to *de novo* synthesis pathway, degradation of sphingomyelin into ceramide by sphingomyelinases is another metabolic pathway which leads to ceramide production, it takes place in the lysosomal membrane and in the cytoplasmic membrane (Hannun and Obeid, 2008). Of note, a mutation in Sphingomyelin phosphodiesterase 1 (also known as acid sphingomyelinase, ASM) causes Niemann-pick disease, characterized by the buildup of toxic amount of sphingomyelin and leading to multi-organ dysfunction (including profound brain damage) (Schuchman and Desnick, 2017).

## Ceramides and brain lipotoxicity

It has been shown that exogenous ceramides could induce hypothalamic lipotoxicity, ER stress and decreased sympathetic tone to the BAT, which leads to decreased thermogenesis and feeding-independent weight gain (Contreras et al., 2014). In addition, genetic modulation of ceramide-induced ER stress pathway in the VMH modulates energy balance by influencing BAT thermogenesis and insulin sensitivity, as well as promoting an overall improvement of the metabolic phenotype of leptin and insulin resistant obese rats (Contreras et al., 2014). In this work, genetic overexpression of GRP78 (the chaperone glucose-regulated protein 78) in the VMH of rats abolishes ceramide action by reducing hypothalamic ER stress and increasing BAT thermogenesis, which lead to weight loss and improved glucose homeostasis. Overall, these data identify a signaling network involving central ceramides, hypothalamic lipotoxicity/ER stress and BAT thermogenesis as a pathophysiological mechanism of obesity. In addition, the amelioration of ER stress by overexpression of GRP78 does no impact ceramide levels in obese Zucker rats, which remain elevated when compared with their lean littermates (Contreras et al., 2017). Therefore, this evidence indicates that ER stress is downstream ceramide's effect (Contreras et al., 2017).

Interestingly, ER stress *per se* could also lead to an increased ceramide synthesis. It has been shown in rodents that ER stress is concomitant with liver insulin resistance and is able to activate SREBP-1c cleavage (Kammoun et al., 2009), and to induce the whole hepatic lipogenic program, thus leading to steatosis and increased ceramide content (Holland and Summers, 2008). Whether a similar mechanism operates in the brain is currently unknown. In addition, it has been shown in peripheral organs that, depending on the ceramide chain length and saturation, the effects could be very different. For example, CerS 1 is mainly involved in the synthesis of C18:0 ceramides, and it has been linked to a greater insulin sensibility in muscle cells, conversely to other CerS isoforms (Frangioudakis et al., 2013). In brain, Zhao et al. reported that Cers1 deficiency dramatically affects sphingolipid homeostasis and leads to Purkinje cell loss, lipofuscin accumulation and overall functional deficit in mice (Zhao et al., 2011; Ginkel et al., 2012).

## A specific role for CPT-1c in brain ceramide metabolism?

Recently, the brain specific isoform of carnitine palmitoyl-transferase, CTP-1c, has been involved in ceramide metabolism and suggested to be a potential downstream effector of leptin action on the control of feeding (Gao et al., 2011). As it has been already demonstrated, leptin inhibits AMPK in the ARC, thus leading to ACC activation and increased malonyl-coA levels (Minokoshi et al., 2004). Gao et al. suggested that CPT-1c, located in the ER, could be a downstream target in the mediation of malonyl-CoA's anorectic signaling action: malonyl-CoA could inhibit CPT-1 to reduce ceramide *de novo* biosynthesis, or it could interact with another target to decrease ceramide level (Gao et al., 2011). Fine molecular studies demonstrated that CPT-1c had a very weak acyl-transferase activity (20–300 times less than CPT-1a and−1b) and preferentially used palmitoyl-CoA as substrate (Sierra et al., 2008). In addition, a significant portion of CPT-1c is localized in the ER. Taken together these data lead to the hypothesis that CPT-1c is involved in ceramide metabolism. Consistently, Gao et al. demonstrated that CPT-1c overexpression in ARC lead to increased ceramide levels whereas the CPT-1c deletion had the opposite effect, and that ceramide metabolism in the Arc was required for leptin's anorectic actions (Gao et al., 2011).

Recent evidence shows that ghrelin (a stomach-derived orexigenic hormone) induces hypothalamic AMPK activation, which decreases ACC activity, reducing malonyl-CoA concentration and therefore releasing inhibition of CPT-1c (Ramirez et al., 2013). CPT1c activity—as explained before—promotes elevated ceramide synthesis and accumulation, which elicits *agrp* and *npy* gene expression and subsequently hyperphagia. Interestingly central inhibition of ceramide synthesis with myriocin negates the orexigenic action of ghrelin through the normalization of orexigenic neuropeptide levels, pointing out a direct role for hypothalamic ceramides in the control of food intake (Ramirez et al., 2013). The authors further demonstrate that CPT-1c is required to mediate the anorectic action of leptin in mice, and that both CPT-1c and ceramide downregulation in hypothalamus are specifically required for the malonyl-coA anorectic action (Gao et al., 2011).

## Lipid metabolism in other brain regions contributes to the regulation of energy homeostasis

Besides the hypothalamus, other brain areas have been shown to be involved in the regulation of energy homeostasis. Regarding food behavior, satiation signals arising in the gastro-intestinal (GI) system converge on the dorsal hindbrain and are integrated with taste and other inputs (Schwartz et al., 2000; Woods, 2009). The dorsal hindbrain connects directly with the ventral hindbrain, where neural circuits direct the autonomic nervous system to influence blood glucose, and where the motor control over eating behavior is located (Woods and D'Alessio, 2008). The hypothalamus and other brain areas, such as hippocampus and striatum, integrate satiation, adiposity and nutrient signals with time of day and other factors like experience, social situation, and stressors. Once integrated, output signals regulate feeding behavior (including food preference, hedonic behavior), motivation (to search food), learning as well as energy expenditure or glucose homeostasis (Woods and D'Alessio, 2008; Woods, 2009). The hippocampus itself is described as a regulator of feeding behavior and body weight regulation (Davidson et al., 2007). Recently, Picard et al. demonstrated that a decreased TG-hydrolysis in hippocampus, through pharmacological or genetic inactivation of lipoprotein lipase (LPL), lead to obesity in both rats and mice (Picard et al., 2014b). In addition, data shows that obesity-associated cognitive impairment could be improved by selectively lowering TG, while intracerebroventricular (ICV) injection of triolein impairs learning in normal mice (Farr et al., 2008). Taken together, these observations raise the possibility that nutritional lipids, and particularly TG, could directly affect the encoding of reward in the mesocorticolimbic system (Farr et al., 2008). Indeed, TG processing enzymes and lipoprotein receptors are expressed in the brain, and several lines of evidence indicate that circulating TG-rich particles access the brain (Wang and Eckel, 2012).

The intra-hippocampal LPL inhibition leads to increased body weight due to decreased locomotor activity and energy expenditure but with no change in food intake, concomitant with high parasympathetic tone (Picard et al., 2014b). Interestingly, Magnan and colleagues identified *de novo* ceramide biosynthesis as a potential molecular mechanism by which altered hippocampal TG hydrolysis may affect energy balance. Ceramide content is increased upon LPL inhibition, and pharmacological inhibition of the *de novo* ceramide biosynthesis pathway is sufficient to prevent body weight gain and the associated phenotype in these animals (Picard et al., 2014b).

Recently, Cansell et al. (2014) showed that chronic brain TG delivery rapidly reduced both spontaneous and amphetamine-induced locomotion, abolished preference for palatable food, and reduced the motivation to engage in food-seeking behavior. Conversely, targeted disruption of the TG-hydrolyzing enzyme LPL specifically in the nucleus accumbens (area involved in cognitive processing of aversion, motivation and reward) increased feeding and food seeking behavior. Prolonged TG perfusion resulted in a return to normal palatable food preference despite continued locomotor suppression, suggesting that adaptive mechanisms occur (Cansell et al., 2014). Overall these results firmly establish that central hydrolysis of nutritional TG can be detected by the mesolimbic system through a LPL dependent mechanism, modulate the brain reward system and promote a state of craving for palatable food, and reduced energy expenditure associated with lower physical activity (two core mechanisms in the etiology of obesity). However, the inner mechanism relaying LPL action is not known, and it is likely to consider that, in the absence of exogenous lipids coming from LPL activity, lipogenesis and subsequent ceramide accumulation with ER stress, could be implicated (Weinstock et al., 1997; Wagner et al., 2004) and thus control food preference and reward seeking behavior.

## Ceramide metabolism as a target for metabolic diseases?

A recent study combining lipidomic analysis in mouse models of obesity and in human prospective cohorts evidenced that plasma ceramides were diabetes susceptibility biomarker candidates (Wigger et al., 2017). A deep molecular analysis of the role of ceramide metabolism will help to understand the precise role of these sphingolipids in metabolic disease at the brain levels. As a number of pharmacological targets exists for ceramide reduction in pre-clinical studies, and some medications which inhibit ceramide production are currently approved for human use (Kornhuber et al., 2010), novel therapies targeting ceramide accumulation in brain (and peripheral tissues) may represent the future of obesity management and a better prevention of T2D. In particular ASM inhibitors hold promise for new therapies for Alzheimer's disease and depression, while acid ceramidase inhibitors are studied for cancer therapies review in Kornhuber et al. (2010). Pushing ceramide metabolism toward the synthesis of less harmful lipids, such as Sphingosine 1-phosphate, with the use of sphingosine kinase 1 activators could also represent a new therapeutic approach to counteract lipotoxicity (Bellini et al., 2015).

## Conclusions

In conclusion, recent data evidenced that ceramides accumulation in brain under lipotoxic conditions might play a role on the deregulation of energy balance and lead to food intake disorders, obesity and the associated perturbation of glucose homeostasis (Table 1). Despite this evidence, the extent and consistency of ceramides effects in specific brain areas, and in particular the specificity of action from various ceramide species, needs to be clarified. Therefore, a better knowledge of ceramide action in brain may lead to earlier and more successful diagnoses and therapeutic options for patients suffering of obesity and associated metabolic disorders.

**Table 1:** Summary of the main effects and mediators of central ceramide actions.

**Area of the brain**	**Ceramide modulation**	**Consequences**	**References**
Hippocampus	LPL inhibition increases *de novo* ceramide biosynthesis.	Increased body weight gain, decreased locomotor activity, high parasympathetic tone.	Picard et al., 2014b
Hypothalamus (VMH)	Central ceramide treatment with cell-penetrating C6 ceramides.	ER stress, sympathetic inhibition leading to reduced brown adipose tissue thermogenesis and weight gain.	Contreras et al., 2014
Hypothalamus (ARC)	CPT-1c overexpression increases ceramide levels; CPT-1c decreased ceramide levels.	Ceramide *de novo* synthesis mediates leptin anorexigenic action on feeding, downstream of malonyl-Co1 and CPT-1c.	Gao et al., 2011
Hypothalamus (mediobasal)	Ghrelin elicits a marked increase in C18:0 ceramides.	Ceramide *de novo* synthesis mediates ghrelin orexigenic action.	Ramirez et al., 2013

## Author contributions

All authors listed have made a substantial, direct and intellectual contribution to the work, and approved it for publication.

### Conflict of interest statement

The authors declare that the research was conducted in the absence of any commercial or financial relationships that could be construed as a potential conflict of interest.
